# Schmerzhafte Knoten am Oberarm

**DOI:** 10.1007/s00105-020-04695-z

**Published:** 2020-09-26

**Authors:** Christina Martha Vallant, Daisy Kopera

**Affiliations:** Klinik für Dermatologie, Medizinische Universität Graz, Auenbruggerplatz 8, 8036 Graz, Österreich

## Anamnese

Ein 57-jähriger Mann in gutem Allgemeinzustand wird mit 3 derben druckschmerzhaften Knoten am linken Oberarm in der Ambulanz vorstellig. Die Hautveränderungen hätten sich innerhalb der letzten 4 Wochen entwickelt. Manchmal habe er ein komisches Kribbeln verspürt. Durch gelegentlich auftretenden Juckreiz habe er gekratzt, deshalb finden sich etwa 3 mm haltende festhaftende Krusten im Zentrum jeder Läsion.

## Klinischer Befund

**Figure 1:**
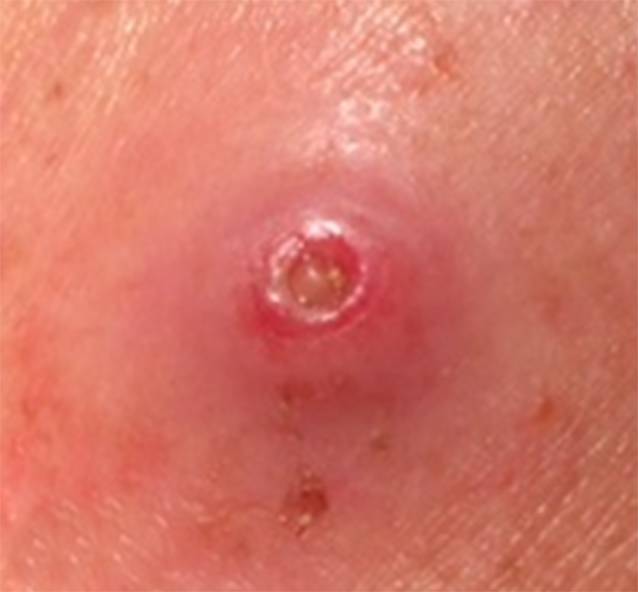

Am linken Oberarm finden sich 3 etwa 5–10 cm voneinander entfernte Knoten. Die unmittelbare Umgebung ist sehr diskret gerötet. Nach Ablösen einer der Krusten zeigt sich im Zentrum des Knotens ein derber gelblicher Pfropf (Abb. 1). Beim Versuch, den Pfropf zu exprimieren, klagt der Patient über starke Schmerzen, sodass ein Lokalanästhetikum zur Anwendung kommt.

## Wie lautet Ihre Diagnose?

## Weiteres Procedere/Therapie

**Figure 2:**
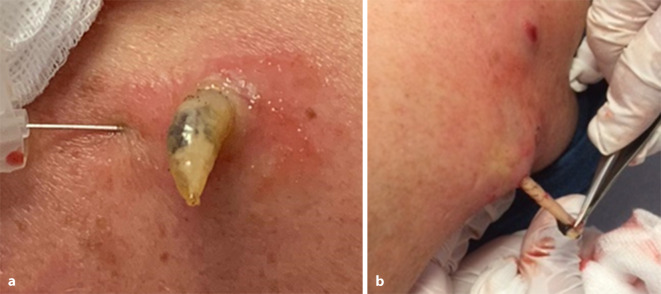

**Figure 3:**
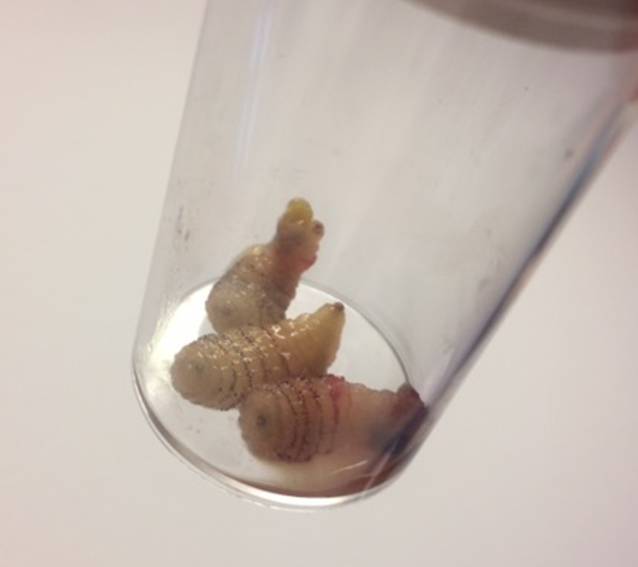

Während der Applikation des Lokalanästhetikums wölbt sich der gelbliche Pfropf immer weiter vor, und es entpuppt sich eine kleinfingerdicke Made (Abb. 2a). Mit weiterem Druck durch das subkutan injizierte Lokalanästhetikum von unten her kann mithilfe einer Pinzette eine 35 mm lange Made entwickelt werden (Abb. 2b). Gleichartig werden die beiden restlichen Läsionen behandelt, auch daraus lassen sich ebensolche Parasiten ziehen, die nach Recherche eindeutig als Larven/Maden einer Dasselfliege identifiziert werden können (Abb. 3).

## Klinische Differenzialdiagnosen

Differenzialdiagnostisch (in alphabetischer Reihenfolge) müssen in Erwägung gezogen werden:

- Arthropodenreaktion,
- Epidermiszysten,
- Furunkulosis,
- Leishmaniose,
- Malignome: Merkel-Zell-Karzinom, kutanes Lymphom, Hautmetastase,
- Pyoderma,
- Sporotrichose,
- Xanthogranulom.

## Definition

Während die afrikanische Tumbufliege (auch Mangofliege, *Cordylobia anthropophaga*), ein Ektoparasit aus der Gattung *Cordylobiae*, ihre Eier auf feuchter Unterlage, auch z. B. auf verschwitzter Kleidung ablegt und die schlüpfenden Larven (= Maden) sich aktiv in die Haut bohren, legt die südamerikanische Dasselfliege (*Dermatobia hominis*) aus der Familie der *Oestridae* ihre Eier auf ein blutsaugendes Insekt, üblicherweise auf Stechmücken oder Zecken, damit diese die Eier auf die Haut von Säugetieren übertragen. Man nennt das Phoresie (von gr. φορείν [phorein] „tragen“), es handelt sich um eine „vorübergehende Transportgesellschaft“. Dort entwickeln sie sich aufgrund der Körpertemperatur des Wirts weiter, und die Larven gelangen durch kleinste oberflächliche Wunden oder Haarfollikel in die Haut, oder die Fliegen legen Eier direkt auf Wunden [1, 2]. Charakteristische Widerhäkchen erleichtern das Eindringen. Die Larven ernähren sich in der Haut von Exsudaten, machen 3 Larvenstadien durch und verlassen dann den Wirt, um sich in der Erde zu verpuppen und zur Fliege weiterzuentwickeln [1, 3]. Mitunter verursachen die heranwachsenden Larven nicht nur ein Kribbeln (durch die sich bewegenden Widerhäkchen), sondern auch furunkuloide Entzündungsherde, auch Dermalmyiasis genannt [4].

Begleitend kann es zu einer bakteriellen, häufig Staphylokokken-induzierten Superinfektion mit Fieber und regionärer Lymphadenopathie kommen, wo der Einsatz eines Antibiotikums erwägenswert ist. Die Therapie der Wahl ist die lokale Entfernung der Larve. In der Literatur werden neben Inzision der Höhle auch andere, kuriose therapeutische Ansätze wie die Auflage von Speck [5] beschrieben. Durch den Okklusionsverband mit einer Scheibe Schweinespeck wird die am Hinterleib sitzende Atemöffnung der Made luftdicht abgedeckt. Um an Luft zu kommen, wandert sie durch den Speck an die Oberfläche und verlässt so atraumatisch die Haut des temporären Wirts. In unserem Fall gelang es durch subkutane Unterspritzung der Larvenhöhlen mit einem Lokalanästhetikum (Abb. 2a), sie durch Druck von unten durch die als Atemöffnung dienende Hautpore atraumatisch und in toto zu bergen (Abb. 2b).

**Diagnose:** Furunkuloide kutane Myiasis

## Diskussion

Denkt man an die steigende Inzidenz der Skabies, so sind Parasitosen in der westlichen, zivilisierten Welt gar nicht so selten. Die Läsionen die durch Larven (Maden) tropischer Fliegen verursacht werden, kommen aber relativ selten vor. Die ersten Differenzialdiagnosen, die bei Betrachtung derartiger Hautläsionen in Erwägung gezogen werden, gehen zumeist nicht in diese Richtung. Es sind jedoch die einfachen Dinge, die uns beim Anblick einer Hautveränderung in den Sinn kommen sollten [6, 7]. Ganz klar war hier die Anamnese unvollständig, denn im Nachhinein erzählte der Patient, dass er vor etwa 5 Wochen von einer Wanderreise durch Costa Rica heimgekehrt sei und dort in feuchtheißem Regenwaldklima gelegentlich auch unter eher abenteuerlichen Umständen übernachtet habe. Kontakt mit diversen Insekten sei keine Seltenheit gewesen.

## Fazit für die Praxis

Es wird empfohlen, bei der Anamneseerhebung sorgsam zu sein und auch Auslandsaufenthalte bzw. Kontakt zur Natur zu erfragen.
